# Rapid radiation in bacteria leads to a division of labour

**DOI:** 10.1038/ncomms10508

**Published:** 2016-02-08

**Authors:** Wook Kim, Stuart B. Levy, Kevin R. Foster

**Affiliations:** 1 Department of Zoology, University of Oxford, Oxford OX1 3PS, UK; 2 Oxford Centre for Integrative Systems Biology, University of Oxford, Oxford OX1 3QU, UK; 3 FAS Center for Systems Biology, University of Harvard, Cambridge, Massachusetts 02138, USA; 4 Center for Adaptation Genetics and Drug Resistance, Department of Molecular Biology and Microbiology, Tufts University School of Medicine, Boston, Massachusetts 02111, USA

## Abstract

The division of labour is a central feature of the most sophisticated biological systems, including genomes, multicellular organisms and societies, which took millions of years to evolve. Here we show that a well-organized and robust division of labour can evolve in a matter of days. Mutants emerge within bacterial colonies and work with the parent strain to gain new territory. The two strains self-organize in space: one provides a wetting polymer at the colony edge, whereas the other sits behind and pushes them both along. The emergence of the interaction is repeatable, bidirectional and only requires a single mutation to alter production of the intracellular messenger, cyclic-di-GMP. Our work demonstrates the power of the division of labour to rapidly solve biological problems without the need for long-term evolution or derived sociality. We predict that the division of labour will evolve frequently in microbial populations, where rapid genetic diversification is common.

## Abstract (web summary)

The division of labour—where individuals specialise on different tasks—is fundamental to many sophisticated and ancient biological systems. Here the authors show that bacteria can deploy a robust and functional division of labour in a matter of days via a single mutation.

## Graphical abstract

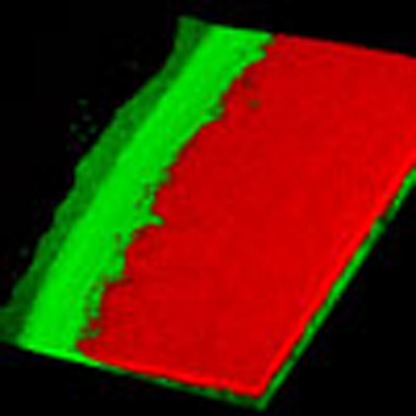

The specialization of individuals on different tasks is central to the organization of both biological and economic systems. The enlightenment philosopher Adam Smith described such specialization in terms of the division of labour; he discussed factory workers performing different tasks along a production line^1^. In evolutionary biology, the division of labour is considered one of the defining features of the major transitions in evolution, which include the origin of genomes, the eukaryotic cell, multicellular organisms and societies^2^,^3^,^4^,^5^,^6^,^7^,^8^. Here, the division of labour is probably best known from the social insects where individual workers perform specific roles for the colony, such as foraging, guarding or nursing^4^. These individual-level behaviours lead to self-organization and the emergence of coordinated colony-level phenotypes that are robust to perturbation^9^,^10^.

At the cellular level, the division of labour emerges during multicellular development where different cells and tissues specialize on different functions. Such development is seen in microbial species that have evolved a differentiation programme, including the slime mold *Dictyostelium discoideum* where cells self-organize in space and generate a multicellular phenotype that both migrates and forms a fruiting body containing a stalk and spores^11^. Different functions also emerge in bacteria during spore formation in *Myxococcus* and *Bacillus* spp. and during heterocyst formation in cyanobacteria^12^,^13^,^14^,^15^,^16^. Finally, species of microbes with different but complementary metabolism have the potential to interact in a way that benefits all parties, such as syntrophic associations of bacteria and archaea^17^. These examples lack the clear collective phenotypes seen in systems like multicellular organisms or spore-forming microbes, but the complementarity of the different species are sometimes considered a ‘metabolic' division of labour^18^.

The canonical examples of the division of labour—the eukaryotic cell, multicellular organisms, derived animal societies—are all associated with long-term evolution. Can the division of labour also evolve rapidly in biological systems? Some solitary insects, like halictine bees, spontaneously generate a division of labour when individuals are forced to share a nest^19^. Such example shows the potential for the rapid emergence of the division of labour, although here it rests upon pre-existing phenotypic responses rather than genetic changes and evolution.

Bacteria evolve and diversify very rapidly, over a period of days in novel environments. This diversification is often explained by ecological adaptation to a new niche. For example, bacteria can rapidly exploit a new niche by attaching to glass and gaining better access to oxygen^20^,^21^. The evolution of diversity can also result from adaptation to a pre-existing genotype including evolving to feed on a factor that a pre-existing genotype produces^18^,^22^,^23^,^24^,^25^, and from the selection of genotypes that confer resistance to antimicrobial compounds^26^. These studies show the evolution of new interactions in microbial populations where the activity of one genotype helps another. However, they do not show, or claim to show, the division of labour where different genotypes work together to create a new collective phenotype. Another potential benefit of the evolution of diversity is bet-hedging. Diversity can enable a particular strain background to survive unpredictable changes in the environment by making at least one variant that can survive, which is known as the insurance hypothesis^27^,^28^,^29^.

Bacteria then are known to rapidly diversify into distinct ecological roles that can interact with one another. Given the obvious benefits of the division of labour described in organisms with defined differential programmes, this begs the question whether rapid radiations also enable different genotype to work together and solve problems collectively. Specifically, can diversification rapidly generate the division of labour and collective phenotypes that are typically associated with long-term evolution and development? To demonstrate the rapid evolution of a division of labour, here we experimentally identify three key properties in an evolving group of bacteria: (i) rapid radiation into distinct genotypes; (ii) the emergence of an integrated collective phenotype that achieves more than the sum of its parts and (iii) the evolution of distinct roles that work together to achieve the collective phenotype. Our work shows that rapid diversification does indeed allow bacteria to make use of the power of the division of labour.

## Results

### Rapid radiation and the evolution of collective spreading

**Figure 1:**
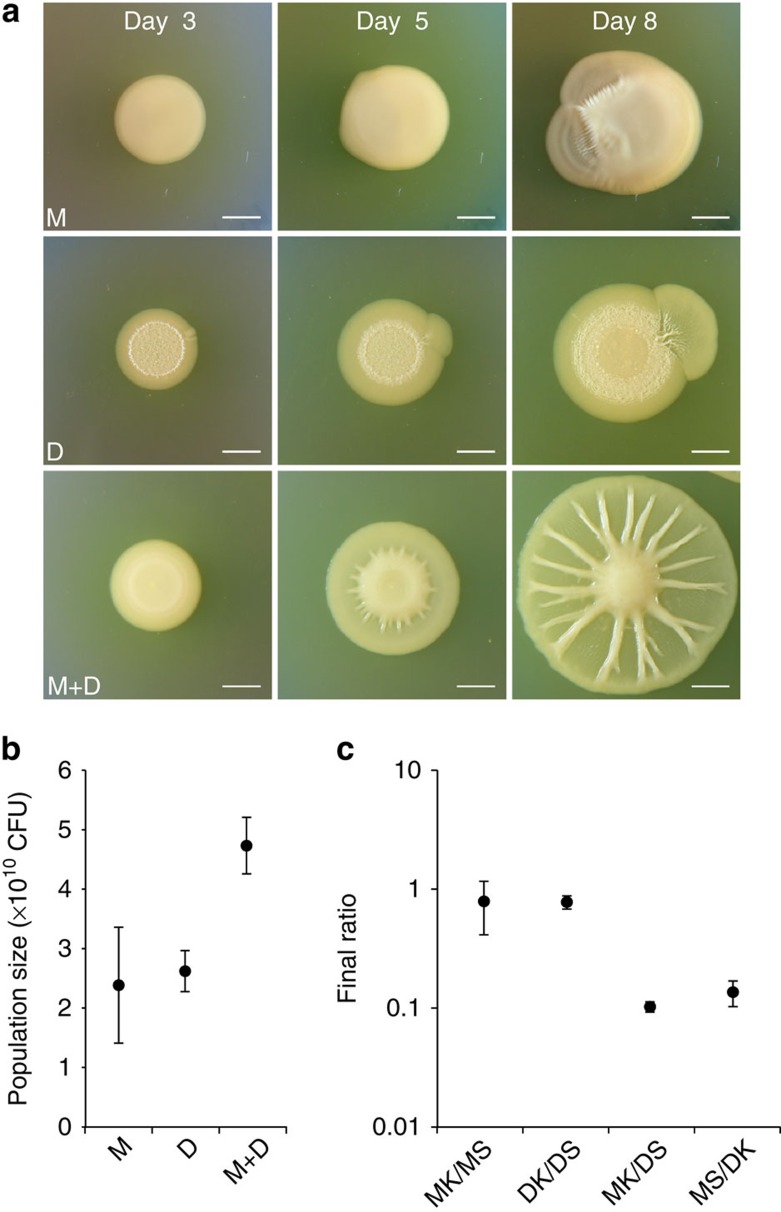
Emergence of the spreading phenotype and comparison of the population size of single and mixed morphotype colonies. (**a**) Spreading fans emerge from a spotted mucoid M colony (top), comprising M and a genetic variant, D, exhibiting a dry colony morphology. The same phenotype emerges from D (middle), and is reproduced by mixing M and D (bottom). Scale bar, 5 mm. (**b**) Estimates of the total population size in colony-forming units (CFU) of single or mixed morphotype colonies on day 8. One-way analysis of variance (ANOVA; *P*=0.000957). Tukey's honest significant difference (HSD): M versus D (*P*>0.05); M versus M+D (*P*<0.01) and D versus M+D (*P*<0.01). (**c**) Ratio of strains in mixed colonies, as indicated on the *x* axis, on day 8. Each strain was engineered to be either kanamycin (K) or streptomycin resistant (S). One-way ANOVA (*P*=0.002824). Tukey's HSD: MK/MS versus DK/DS (*P*>0.05); MK/MS versus MK/DS (*P*<0.05); MK/MS versus MS/DK (*P*<0.05); DK/DS versus MK/DS (*P*<0.05); DK/DS versus MS/DK (*P*<0.05) and MK/DS versus MS/DK (*P*>0.05). All mixed colonies were initiated at a 1:1 ratio. For all experiments, plotted are the means from three independent colonies, and the error bars represent the s.d.

We recently isolated a mucoid strain in colonies of *Pseudomonas fluorescens* Pf0-1 (ref. 30), which we call ‘M' here that gets its mucoidy from a glucose-based polymer (Supplementary Table 1). M harbours a single-nucleotide frameshift mutation in the *rsmE* gene, which encodes a repressor of multiple secretions^30^ (Supplementary Table 2). When plated clonally, we observe that M evolves and repeatedly generates a new phenotype that enables the colonies to spread outwards (Fig. 1a, see Methods for details). Outward fan-like growth is common at the edge of ageing bacterial colonies. However, these fans are typically thought to be a clonal population of a mutant that has acquired the ability to grow or move faster as compared with the parent cells^31^. In contrast, we found that our spreading fans consistently comprised a mixture of two strains: both the original strain M and a new morphotype that we call ‘D' here because of its dry and wrinkly colony morphology. The spreading phenotype is reproduced whenever M and D are mixed, but only appears after a significant delay in pure colonies because, as we will show, it rests upon mutations to generate the other variant (Fig. 1a). The spreading phenotype allows colonies to both gain territory and to produce more cells in way that neither strain achieves on its own (Fig. 1b). These observations were consistent with the *de novo* evolution of a division of labour where distinct types generate a shared phenotype—rapid spreading—that is not possible on their own. We therefore subjected our experimental system to detailed analyses in order to understand how an apparent division of labour could so rapidly evolve from a simple bacterial colony.

### M and D robustly self-organize in space

**Figure 2:**
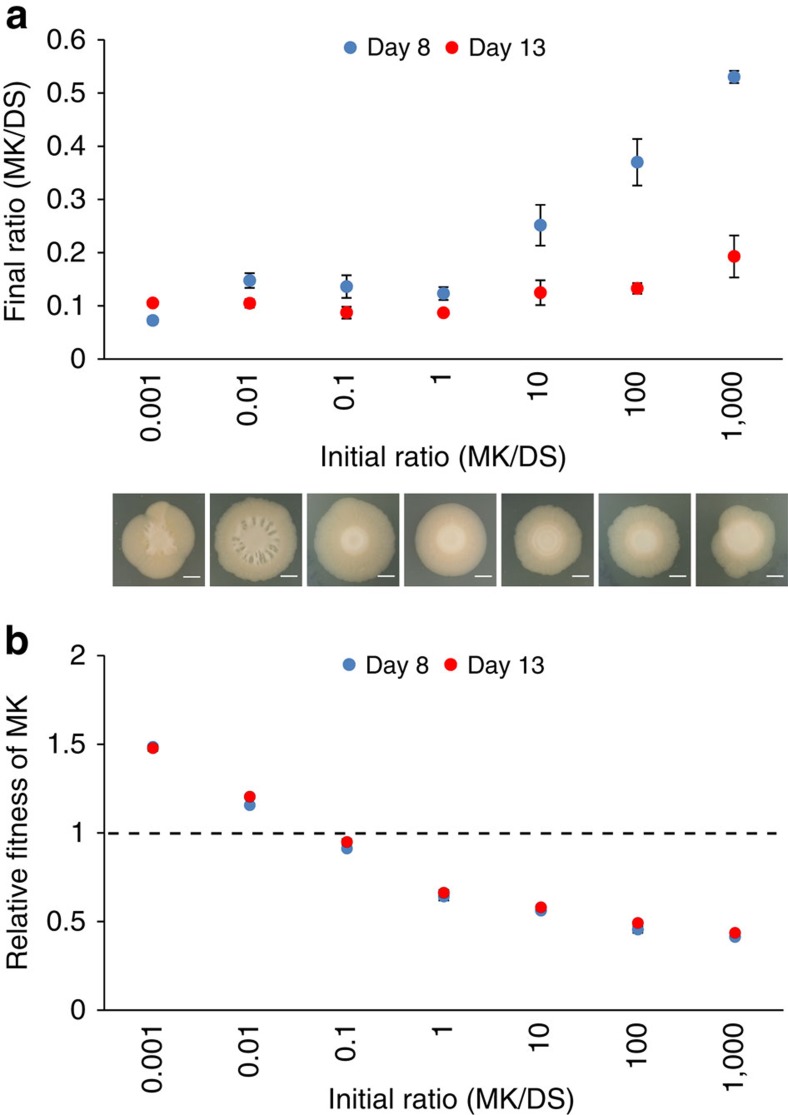
The frequency of M and D morphotypes approach a characteristic ratio independent of the initial frequency. (**a**) The ratio of kanamycin-resistant M (MK) compared with streptomycin-resistant D (DS) in mixed colonies on day 8 (blue) and day 13 (red) plotted against the initial ratio. Shown below are corresponding images of mixed colonies on day 8. Scale bars, 5 mm. (**b**) The data in **a** plotted as relative fitness of MK compared with DS. The dotted line represents equal fitness. All error bars represent the s.d. of the mean from three independent colonies.

**Figure 3:**
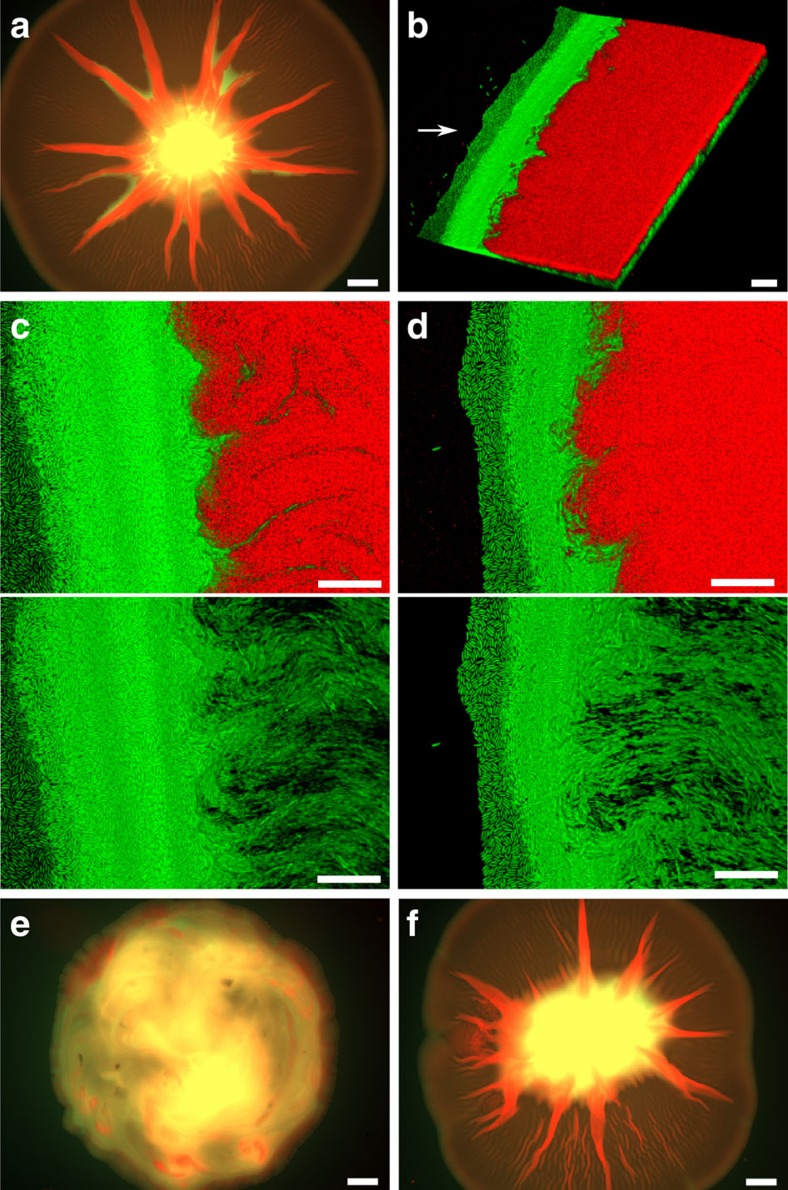
M and D self-organize into characteristic spatial patterns. (**a**) Epifluorescence image of a mixed colony of fluorescently tagged M (green) and D (red) on day 8. (**b**) Corresponding confocal laser scanning microscopy image of the edge. The white arrow points at the edge of the colony. (**c**,**d**) Confocal images of the edge on day 2 (**c**) and day 22 (**d**). The two images within each panel are identical except the red channel has been removed from the bottom images. (**e**,**f**) Day 8 epifluorescence image of a mixed colony disturbed daily from day 3 (**e**) or only once on day 3 (**f**). All mixed colonies were initiated at a 1:1 ratio. Scale bars, 2 mm or 20 μm in epifluorescence and confocal images, respectively. All confocal images are three-dimensional renderings.

To probe the robustness of the phenotype, we started the experiment across an extremely wide range of initial frequencies of M and D that covers several orders of magnitude. Not only did all conditions generate the spreading phenotype, we were surprised to find that the two strains always approach a characteristic ratio over time, ∼10% M to 90% D, irrespective of the starting frequencies (Figs 1c and 2a). Accordingly, we detect strong negative frequency-dependent selection in the system where each strain can outcompete the other when rare (Fig. 2b). The stability in the frequency of the two types is further suggestive of a well-organized collective phenotype. To understand this better, we directly imaged the mixed colonies using epifluorescence and confocal laser scanning microscopy using a metallurgic objective, which allows us to image without using a coverslip that would distort the colony structure. This revealed that the two strains segregate strongly in space: M mostly localizes at the centre, whereas D dominates the spreading bulk of the colony (Fig. 3a) where it sits atop a thin layer of M (Fig. 3b).

The ability of D to sit on top of M is seen before spreading starts and likely explains its ability to initially increase in frequency as cells at the top of a colony gain an advantage by having the best access to oxygen (Fig. 3c)^21^,^30^. However, segregation is most striking at the edge of the colony where the spreading is actually occurring. Here, D again dominates except at the very edge where there is a continuous strip of M, measuring ∼100 μm, that forms the circumference of the entire colony. This thin strip forms within the first 2 days of mixing (Fig. 3c) and remains stable for as long as we run the experiments (22 days, Fig. 3d). The relative positioning of the two strains is crucial. Daily disruption of the mixed colony inhibits spreading (Fig. 3e). When the mixed colony is disrupted only once, however, the two strains self-organize back into their relative positions and spreading ensues (Fig. 3f). This robustness also helps to explain why the strains are co-selected during spreading and the convergence of the two morphotypes on a characteristic ratio (Fig. 2). A specific spatial structuring involving both strains is necessary for both the commencement and maintenance of the collective phenotype.

### D and M carry out distinct tasks to spread collectively

How are the two strains interacting to enable spreading? One possibility is that D is passively supplying a factor—a signal or metabolite—that promotes the motility of M cells. However, time-lapse confocal microscopy suggests instead that D pushes M along at the edge of the colony (Supplementary Movie 1), because narrow tracks of stationary M cells are seen underneath the moving D cells akin to scraped earth beneath the base of a moving glacier (Supplementary Movie 2). Furthermore, pure D colonies exhibit a wrinkly morphology (Fig. 1a), which has been shown to be associated with the buildup of compressive forces that accumulate through individual cell division events within colonies that produce adhesive extracellular polymers^32^. Consistent with their adhesive nature, the dry-wrinkly D colonies expand slower in comparison to the smooth M colonies (Fig. 1a). Moreover, this wrinkly morphology is greatly reduced in the mixed morphotype colonies (Fig. 1a), suggesting that the forces generated by D are released in the form of pushing when interacting with M.

To further support the importance of D pushing M, we sought an experimental test of this role of D. We reasoned that if D is pushing M and the edge of the colony outwards, then physically removing the cells between the edge and the centre of the colony, where D occupies the bulk of the space (Fig. 3, [Fig f4]), will slow colony spreading. We performed this test and found that spreading is indeed inhibited. However, the same treatment does not deform the shape of a colony without the spreading phenotype (Supplementary Fig. 1a). This demonstrates that the treatment does not impact the basal level of cell division-driven colony expansion. In sum, multiple lines of evidence support the model that D pushes the edge along, and we provide further evidence below.

**Figure 4 f4:**
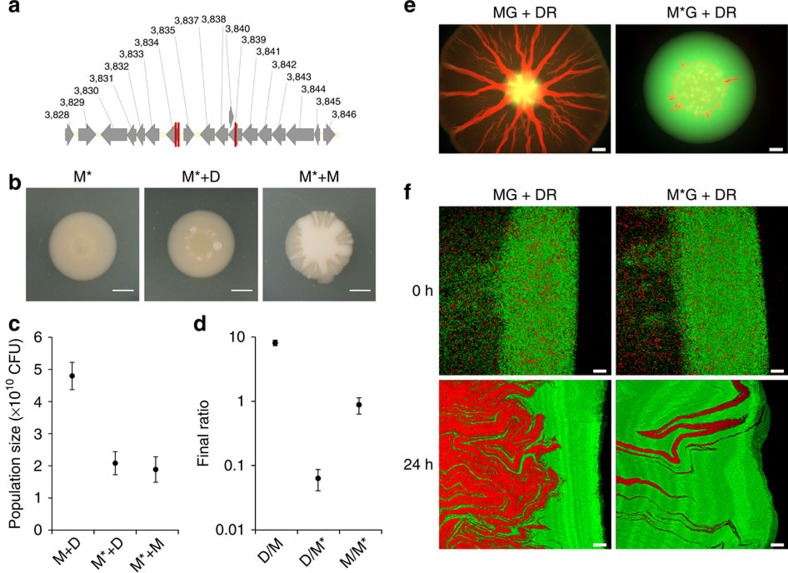
The mucoid polymer produced by M is required for both spreading and spatial structuring. (**a**) A schematic of the gene cluster associated with mucoid polymer production in M. The numbers represent the annotated Pfl01_ORF designation, and the red vertical bars indicate the sites of transposon insertions that result in the loss of the mucoid phenotype in M. (**b**) Colony morphology of non-mucoid M* (MΔ*Pfl01_3834*) on its own and in mixture with D or M on day 8. Scale bars, 5 mm. (**c**) Estimates of the total population size of various mixed colonies on day 8. One-way analysis of variance (ANOVA; *P*=0.000167). Tukey's honest significant difference (HSD): M+D versus M*+D (*P*<0.01); M+D versus M*+M (*P*<0.01) and M*+D versus M*+M (*P*>0.05). (**d**) Day 8 strain ratio in mixed colonies as indicated on the *x* axis. D is streptomycin-resistant and M and M* are kanamycin-resistant. One-way ANOVA (*P*<0.0001). Tukey's HSD: D/M versus D/M* (*P*<0.01); D/M versus M/M* (*P*<0.01) and D/M* versus M/M* (*P*>0.05). (**e**) Epifluorescence images of mixed colonies of fluorescently tagged M (green, MG) or M* (green, M*G) and D (red, DR) on day 8. (**f**) Mucoidy in M is needed for characteristic spatial structuring of the two morphotypes. Three-dimensional rendering of confocal images of the edge of mixed colonies after spotting (0 h) and 24 h later. For all experiments, plotted are the means from three independent colonies, and the error bars represent the s.d. All mixed colonies were initiated at a 1:1 ratio. Scale bars, 2 mm or 20 μm in epifluorescence and confocal images, respectively.

Why is M required for the spreading phenotype? On their own, M colonies expand faster than D colonies, but this is dramatically enhanced when D and M are mixed together (Fig. 1a). Based on the mucoid appearance of M, we predicted that a mucoid polymer secreted by M could function as a lubricant. More formally, we hypothesised that that the polymer acts as a wetting agent that hydrates the colony and reduces the resistance to movement (viscous flow), which allows D to push M along from behind. To address this, M was subjected to random transposon mutagenesis to disrupt the production of the polymer. This identified three independent transposon insertions within a cluster with homologies to extracellular polysaccharide biosynthetic genes (Fig. 4a). Deleting one of the targeted genes in the M background (M*) also removed mucoidy (Fig. 4b). Moreover, the M* strain that lacks the polymer does not evolve the spreading phenotype (Fig. 4b), and pre-mixed colonies of M* and D do not show spreading (Fig. 4b,e). The M*+D mixture increases in population size at a rate that is comparable to the pure cultures and other mixtures that do not exhibit the spreading phenotype (Figs 1b and 4c). The loss of spreading is also associated with a loss of the characteristic spatial structuring. Without the mucoidy in M, the two strains remain relatively well mixed (Supplementary Fig. 2) and D fails to reach the colony edge and dominate the colony (Fig. 4f). D also reaches a much lower population size when mixed with M* relative to being mixed with M (Fig. 4d).

**Figure 5:**
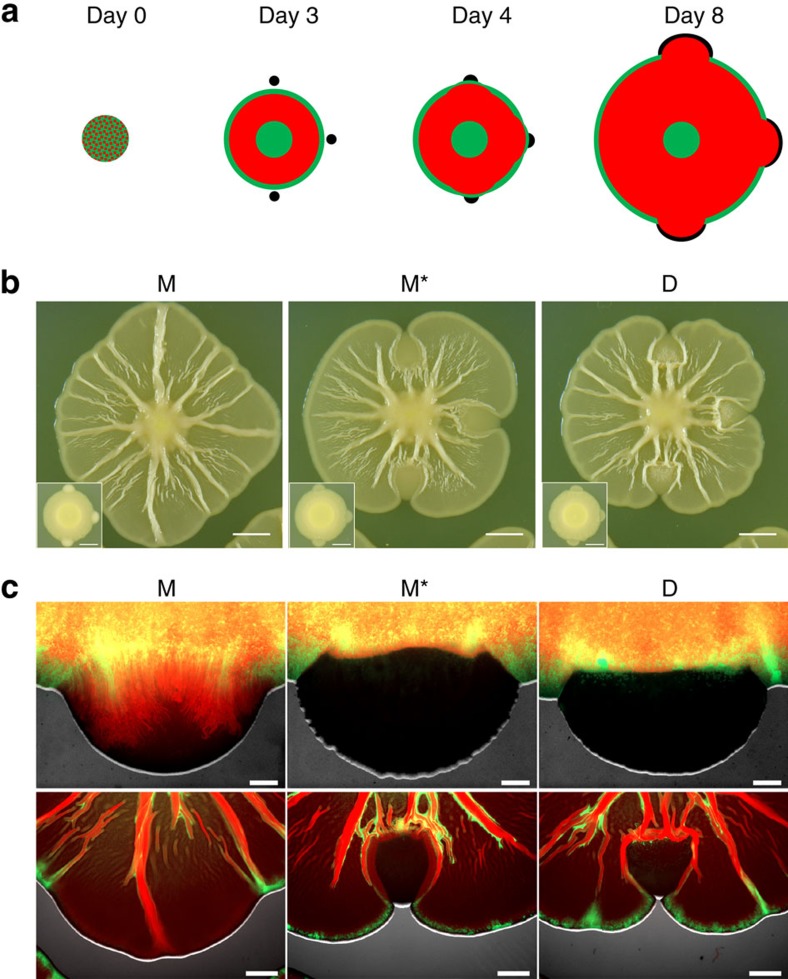
M functions as a lubricant allowing D to push from behind. (**a**) Schematic of the spatial structure construction experiment. Fluorescently labelled M (green) and D (red) cells are mixed 1:1 and spotted. M and D self-organize and spatially segregate, establishing the characteristic spatial structure. Droplets of unlabelled M, M* (no mucoid polymer) or D cells are then placed near the edge of the spreading front on day 3 and visualized on days 4 and 8. (**b**) Spreading continues when the mixed colony encounters a droplet of M, but not M* or D. Main images were captured on day 8 and the inset images show the spatial arrangements on day 4. Scale bars, 5 mm. (**c**) The red-labelled D strain streams into the droplet of cells and pushes the colony edge along, but only when the droplet cells are the mucoid M strain. Epifluorescence images on day 4 (top; 0.5 mm scale) and day 8 (bottom; 2 mm scale). D continues to push through M, but not M* or D. These images correspond to the bottom droplets shown in **b**. See also Supplementary Fig. 3 for more detail on the key treatment where D streams into mucoid M.

We have shown that the mucoid polymer of M is central to the spatial organization of D and M, which ultimately allows D to drive the spreading phenotype (Figs 3 and 4). But does mucoidy also have a lubricating role as we first hypothesized? To examine this, we artificially created the spatial structure that emerges by self-organization of D and M in mixed colonies (Fig. 3). We did this by spotting either M or M* cells at the edge of pure D colonies. Although the correct spatial structure is present whether M or M* is added, D is able to spread when M is provided at the edge but not when M* is added that lacks the polymer (Supplementary Fig. 1b). This suggests that a lubrication effect of the polymer may indeed be important. However, as the spatial structure we created using pure cultures is crude compared with the one that emerges naturally in mixed colonies, we carried out an additional manipulation. This experiment has a more complex design but arguably provides the strongest evidence for both lubrication by M and pushing by D in one go. Here, we prepared mixed colonies of fluorescently labelled M+D cells that, as always, proceeded to naturally self-organize and establish their characteristic spatial structure with M at the front and D behind. On day 3, we then added a droplet of unlabelled cells of either M, M* or D at the edge of the expanding colony (Fig. 5a) in order to test if the colony could continue spreading in the face of this potential barrier. The results of this experiment are clear: only when M is placed at the edge can the colony continue to spread unhindered (Fig. 5b). Moreover, epifluorescence imaging shows that it is the D cells from the original colony that stream into the M droplet and re-establish the spreading phenotype with the unlabelled M cells from the added droplet (Fig. 5c and Supplementary Fig. 3). The observation that the D cells, and not the M cells, continuously stream into the droplet lends further support to the role of D as the strain that generates the pushing force for spreading.

We observe then that the mucoid polymer does indeed act like a lubricant because the spreading colony is blocked by the addition of a droplet that lacks the polymer. An alternative model is that the mucoid polymer of M acts as a signalling or nutrient source to induce spreading in D cells. However, this model would predict that D cells are still fed (or signaled to) by the accompanying M cells as they encounter the M* or D droplets. In this case, therefore, we should expect that the labelled D cells enter all droplets but, in the absence of the mucoid polymer, they would gradually slow down and stop moving somewhere within the droplet. However, this is not observed. Instead, both M* and D droplets appear to create an immediate physical barrier that generates a sharp border against the incoming M+D population (Fig. 5c). In sum, these experiments explain why M and D are both necessary for the spreading phenotype. M makes a polymer that is important for both the emergence of spatial structure and as a lubricant, and D cells are needed to push the edge of the colony outwards.

### Genetic basis of the evolution of the D morphotype

**Figure 6:**
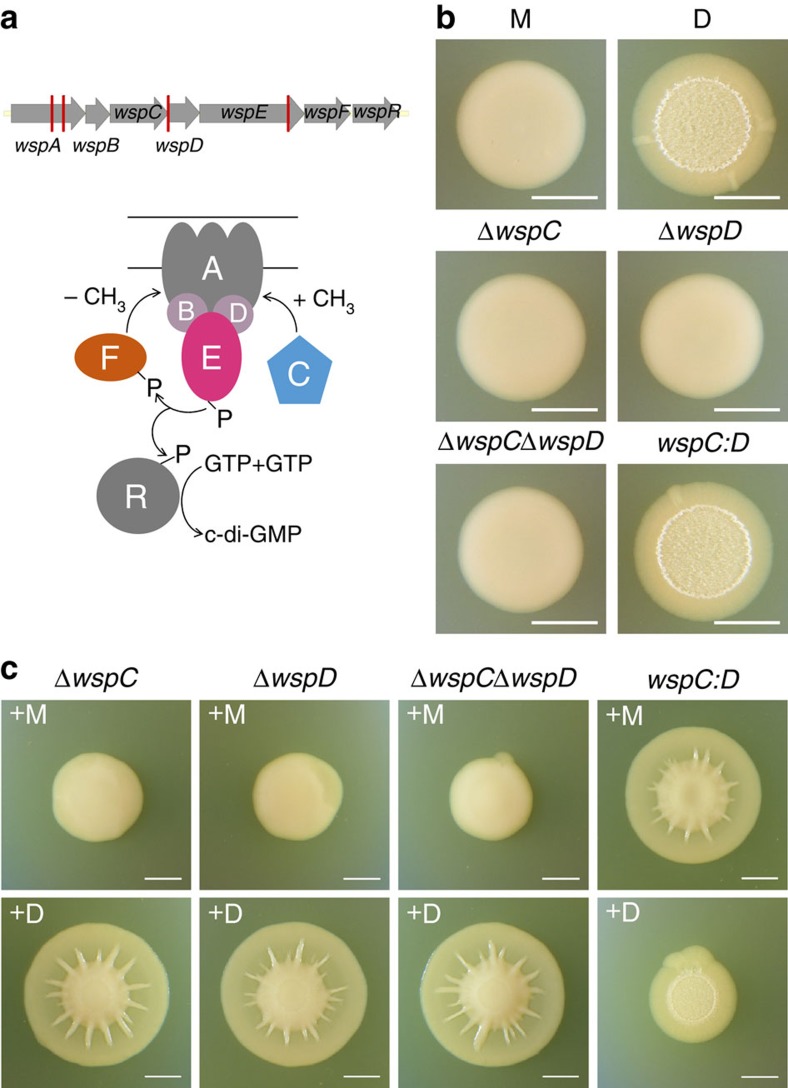
Genetic analyses of the D morphotypes and the spreading phenotype. (**a**) Schematic of the *wsp* operon (top). Red vertical bars indicate the site of individual mutations found among the D morphotypes. A simplified model of the corresponding Wsp signalling system modulating c-di-GMP production (bottom). (**b**) Genetic analysis suggests that the original D strain has a mutation that hybridizes the WspC and WspD proteins. Comparison of the colony morphology of the engineered strains to M and D on day 3. Only the *wspC:D* hybrid mutant (that is, the same mutation found in D) exhibits the same dry morphology as D. (**c**) Comparison of the spreading phenotype of the engineered strains mixed at a 1:1 ratio with either M or D on day 8. Scale bars, 5 mm.

Our experiments document the evolution of a phenotype that is generated by two morphotypes working together and performing distinct roles. This shows that rapid diversification in bacteria can indeed allow them to make use of the division of labour. Rapid evolution, however, typically rests upon very few mutations. How then can bacteria evolve a well-organized, and robust, collective phenotype with only the time for a handful of mutations? In order to answer this question, we sequenced the genome of the D strain, which revealed a two-nucleotide deletion at the tail end of the *wspC* gene (Supplementary Table 2). This mutation places the downstream *wspD* gene within the same reading frame as *wspC*. Wsp proteins function together as a signal transduction system that responds to growth on surfaces (Fig. 6a)^20^,^33^,^34^,^35^: methylation of WspA triggers a phosphorelay to activate WspR, a diguanylate cyclase, which catalyses the formation of cyclic di-3′,5′-guanylate (c-di-GMP) from two molecules of GTP. c-di-GMP is a universal secondary messenger molecule in bacteria, which impacts diverse physiological processes^36^. The Wsp system has been demonstrated to modulate c-di-GMP production in *P. fluorescens*^37^, and specifically in our strain background^38^. Wsp mutants are well known to emerge in *P. fluorescens* in liquid cultures where the phenotype allows cells to stick to the edge of glass culture vessel and form a mat across the liquid surface^20^,^21^. Although the functional outcome is different to our system, the prior observation of *wsp* mutants in experimental evolution made them a particularly promising general candidate for our D morphotype.

Introduction of the same two-nucleotide deletion in M (*wspC:D*) reproduces the same D morphology. However, we found that deleting each gene, or both, fails to create the D morphology (Fig. 6b). Furthermore, the *wspC:D* hybrid mutant reproduces the spreading phenotype when mixed with M, whereas the other mutants require the presence of D to spread (Fig. 6c). The D morphotype then appears to be the product of the function of the WspCD hybrid rather than that of loss of function of either or both proteins. Given that WspC is a constitutive activator and WspD is a scaffolding protein that binds to WspA (Fig. 6a), we predict that the WspCD hydbrid increases methylation of WspA and drives up the production of c-di-GMP. In addition to the wrinkly colony morphology, which results from the production of structurally rigid extracellular polymers in *P. fluorescens* SBW25 and many other species^36^,^39^, motility is also a strongly conserved phenotypic indicator of c-di-GMP production^36^,^40^. Increased c-di-GMP production has been explicitly demonstrated to reduce motility in our specific strain background^38^, and flagella synthesis has been shown to be repressed directly by c-di-GMP in *P. aeruginosa*^41^. Further consistent with the role of c-di-GMP in our phenotypes, both the original D strain and *wspC:D* hybrid mutant display the wrinkly colony morphology (Fig. 6b) and impaired motility (Supplementary Fig. 4). In sum, we observe a mutation in the Wsp system, which is predicted to increase c-di-GMP production, and the resulting wrinkly morphology and reduced motility phenotypes are both the expected effect of increased c-di-GMP.

**Figure 7:**
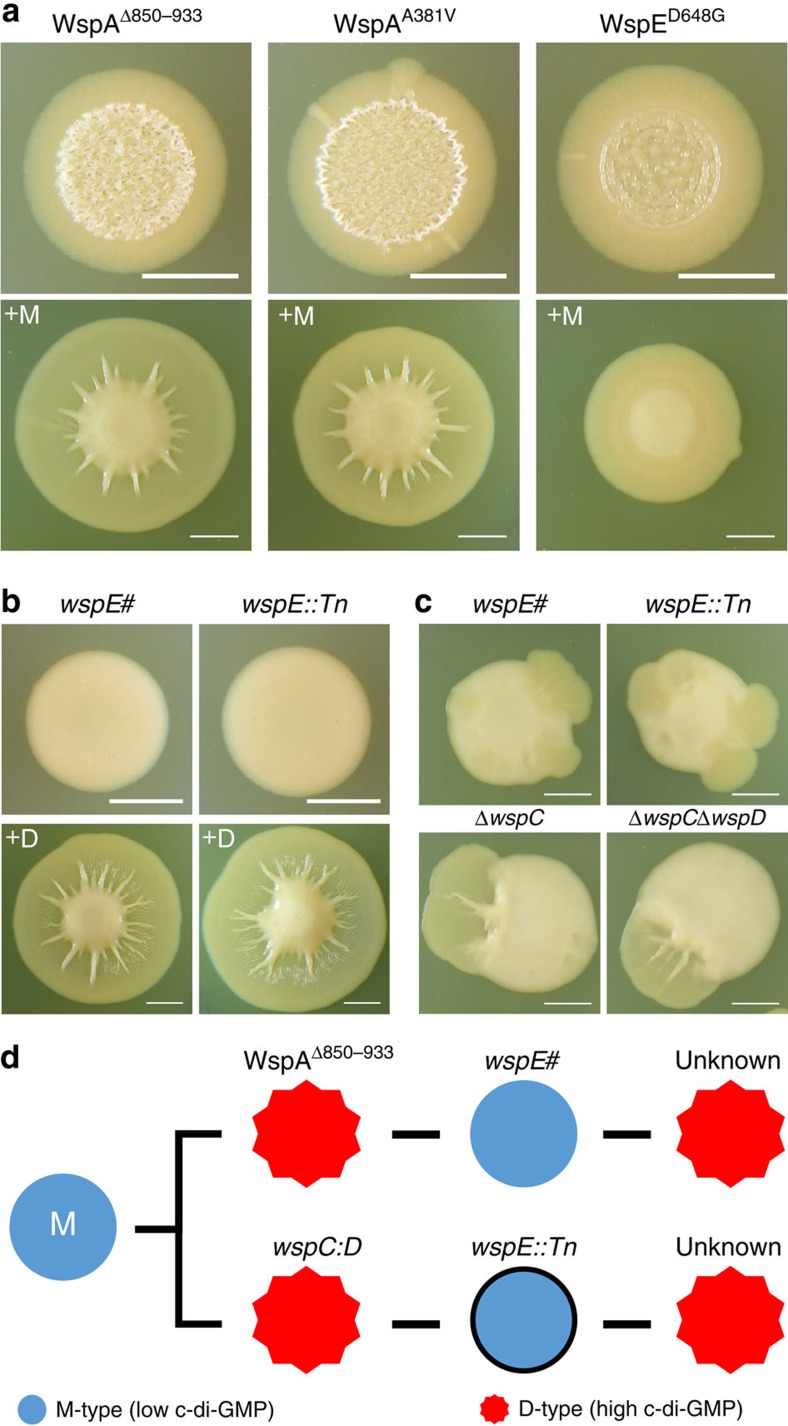
Multiple D genotypes evolve and bidirectional evolution generates M from D and vice versa. (**a**) Phenotypes of additional D morphotypes individually isolated from a single spreading fan emerging from discrete M colonies (day 3, top). Each variant reproduces the spreading phenotype when mixed with M (day 8, bottom). (**b**) Mucoid colony morphology of the *wspE*# and *wspE::Tn* revertants (day 3) and their spreading phenotype when mixed with D (day 8). (**c**) Emergence of spreading fans from various mutants captured on day 9. Each starting strain harbours a mutation predicted to terminally shut down the Wsp signalling pathway, suggesting that additional c-di-GMP production pathways are involved in the transitions between the M and D morphotypes. (**d**) Summary of the bidirectional evolution of M and D morphotypes. All strains of the D morphotype exhibit reduced motility, indicative of reduction in c-di-GMP production. All mutations were naturally selected with the exception of *wspE::Tn* (black outline). All mixed colonies were initiated at a 1:1 ratio. Scale bars, 5 mm.

Individual spreading fans that emerge from M colonies always contain a variant of a single dry-wrinkly morphology in addition to M. Do the *wsp* loci then serve as a common mutational target for the evolution of the D morphotype? Sequencing the *wsp* operon in several independently evolved D morphotypes revealed that all had a single mutation in a *wsp* locus (Figs 6a and 7a, Supplementary Table 2, see Methods). This was again associated with both the wrinkly colony morphology (Fig. 7a) and reduced motility phenotype (Supplementary Fig. 4), suggesting increased c-di-GMP production in all strains that we tested. The most frequent example is a missense mutation in *wspE* (WspE^D648G^), which encodes a phosphorelay protein that activates WspR (Figs 6a and 7a). WspE harbours both histidine kinase and receiver domains. The receiver domain is highly conserved in bacteria and work from the homologous CheY of *Escherichia coli* shows that the specific D648G mutation that we observe can activate the protein in a phosphorylation-independent manner when accompanied by two additional missense mutations^42^,^43^. Moreover, recent studies show that the diguanylate cyclase activity of WspR in *P. aeruginosa* is also not exclusively dependent on phosphorylation^34^,^44^. This suggests that WspE^D648G^ may stimulate WspR or other diguanylate cyclases through another mechanism that does not depend on phosphorylation.

The remaining adaptation events occurred through a mutation in *wspA*: an in-frame deletion which removes 28 amino acids or a missense mutation (WspA^A381V^; Fig. 7a and Supplementary Table 2). Methylation of WspA stimulates the histidine kinase activity of WspE, which in turn activates WspR (Fig. 6a)^35^. This suggests that these WspA mutations stimulate WspE and ultimately WspR to amplify the production of c-di-GMP. All mutations generate a similar colony morphology as the original D strain, with small variations, and each of the independently derived dry morphotypes reproduce the spreading phenotype when mixed with M (Fig. 7a). Moreover, none of the other mutations are predicted to result in loss-of-function of the encoding protein. Instead, they all indicate that the evolution of the D genotype is associated with the activation of the Wsp system and an increase in production of c-di-GMP; the Wsp system is in a low-activity state in M, and the mutations are selected to stimulate it (Supplementary Fig. 4).

### Bidirectional selection of D and M morphotypes

Natural selection repeatedly targets the *wsp* operon and generates the spreading phenotype in M colonies. In addition, the spreading phenotype also repeatedly evolves from pure D colonies (Fig. 1a). Here, the spreading fan always comprises both D and a new morphotype that appears identical to M, and mixing the two strains reproduces the spreading phenotype. Given that specific mutations within the *wsp* operon could act to either stimulate or dampen the signalling pathway (Fig. 6a), we hypothesized that subsequent mutations in *wsp* loci are responsible for the bidirectional selection of D and M morphotypes. We found this to be the case. Resequencing the *wsp* operon in one of the M revertants (*wspE#*) revealed a nonsense mutation in *wspE* that is predicted to shut down the Wsp system (Figs 6a and 7b and Supplementary Table 2). In addition, we undertook a transposon mutagenesis screen in the D background to isolate mutants that produce the M morphology, which isolated a single mucoid mutant (*wspE::Tn*) where the transposon had inserted into the *wspE* gene (Fig. 7b and Supplementary Table 2). As with the original M strain, mixing the M-revertant or the mucoid transposon mutant with D reproduces the spreading phenotype (Fig. 7b). Furthermore, in addition to the loss of the wrinkly colony morphology, both strains regained motility (Supplementary Fig. 4), in accordance with the predicted reduction in c-di-GMP production.

Mutations that first activate and then suppress the Wsp system allow the strain to evolve first from M to D and then back to M (Fig. 7d). However, the last class of mutations work by inactivating the Wsp system and make its reactivation extremely unlikely because this would rest upon reversion and/or restoration of function in the affected proteins (Fig. 6a). This begs the question of whether these strains would be capable of generating the spreading phenotype by again evolving from M to D. To test this, we started colonies using a number of evolved, or engineered, M strains that have a dysfunctional Wsp system. We find that the spreading phenotype re-evolves in each case, where a new D morphotype is once again co-selected (Fig. 7c). This suggests that natural selection for the D morphotype has extended beyond the function of the Wsp system. Consistent with this, previous work has identified at least 38 proteins in our strain background that may harbour a diguanylate cyclase activity^38^, which provides a large set of mutational targets to evolve D from M, and vice versa. Accordingly, every new D morphotype exhibits reduced motility (Supplementary Fig. 5), suggesting c-di-GMP production is changed once again in the evolutionary sequence from D to M, then back to D. Although the underlying genetic effects are diverse, therefore, we observe robust bidirectional evolution that reliably generates whichever partner is missing for collective spreading.

## Discussion

Our work shows that rapid diversification in microbes is a route to the division of labour and an effective collective response to environmental challenges. Evolution in our system starts with a simple diversification that is independent of the division of labour. The D genotype evolves and increases in frequency, presumably due to its ability to sit atop the M strain and gain better access to oxygen (Fig. 3)^21^,^30^. Once D approaches a critical frequency, it is then able to combine forces with the M cells and together they spread. This interaction leads to strong negatively frequency-dependent natural selection, where both types are co-selected and maintained for as long as the experiment is run. Although the evolutionary origin is very different, our experiments show that the interaction between the M and D genotypes shares key similarities with canonical examples of the division of labour, such as those in insect societies and multicellular development. Specifically, we see distinct roles within a social group that work together to generate a collective phenotype. This raises the possibility that the evolution of the division of labour may commonly occur in diversifying microbial communities. In support of this notion, there are a large number of examples where polymorphism, cell–cell interactions and ecological diversification rapidly evolve in bacteria, archaea and yeast^18^,^20^,^21^,^22^,^23^,^24^,^25^,^27^,^28^,^45^,^46^,^47^,^48^. These systems then are good candidates to experimentally test, as we have here, whether different cell types will work together to generate collective phenotypes.

Robustness is a key feature of the division of labour in insect societies^9^,^10^ and multicellular development^49^. We also find that the spreading phenotype is robust to multiple types of manipulation. It will emerge across a wide range of initial frequencies and can rapidly recover from physical disruption. In addition, the system will evolve from multiple M or D genotypes. The robustness and organization of the collective phenotype contrasts with the simplicity of its origin. A single mutation is sufficient to generate D from M, or vice versa. Moreover, all the mutations appear to function via a single intracellular messenger, c-di-GMP. This suggests that the division of labour can be achieved by simply changing the levels of a single intracellular messenger c-di-GMP. C-di-GMP typically functions to trigger the physiological transition from a motile (that is, free-living) to a sessile state (that is, aggregative)^36^. Accordingly, *wsp* genes and others that modulate c-di-GMP production are frequently targeted by natural selection for better attachment and colonization of various solid substrates^20^,^25^. In our system, the upregulation of c-di-GMP production appears to have been co-opted for a quite different function; the ‘sessile' state seen in D is actually the key to colony spreading.

We reliably observe a reverse evolutionary process whereby the spreading phenotype emerges from the D strain. As in the initial case, the spreading fan comprises both D and a new morphotype that appears identical to the original M strain, and experiments where the two strain are plated together again reproduce the spreading phenotype. The bidirectional nature of the evolutionary process provides further support that the division of labour can be potentially achieved by simply changing the levels of a single intracellular messenger c-di-GMP. We even see that the D phenotype will emerge from M strains where the possibility of evolving via the Wsp system is extremely unlikely due to loss-of-function mutations in essential proteins of the Wsp system. Natural selection then finds multiple ways to rewire c-di-GMP regulation and generate the two partners required for collective spreading in our experiments.

The spreading phenotype that we observe shares functional similarities to an established division of labour in *B. subtilis*^16^, which appears to have evolved multiple examples in its long evolutionary history. But how similar is our system to the canonical examples of the division of labour like the eukaryotic cell, multicellular organisms or derived insect societies^2^,^3^,^4^,^5^,^6^,^7^,^11^,^12^,^13^,^15^? Some examples of the division of labour are generated phenotypically from a common genetic background, as occurs in differentiation in multicellular organisms. In this regard, our system is more similar to examples like the endosymbiosis that generated the eukaryotic cell where the partners are genetically different^5^. However, not all mutualisms between genetically different individuals involve the division of labour. Many mutualisms, such as plants and pollinators, are better understood in terms of trade between partners^50^ and lack the collective phenotype that occurs in our system. Moreover, the spreading phenotype displays the spatial structure, self-organization and robustness that is characteristic of many examples of the division of labour, including those in insect societies^9^,^10^. Our work shows that evolving microbes can rapidly employ the division of labour to overcome environmental challenges without the need for social complexity or long-term evolution.

## Methods

### Bacterial strains and growth conditions

*P. fluorescens* strains used in this study are described in Supplementary Table 2. Strain Pf0-1MV (referred to as ‘M' in the main text) is the parent strain of all evolved dry-wrinkly variants. A single isolate exhibiting the dry-wrinkly morphology was randomly chosen as the prototype and designated as ‘D'. Cloning was carried out in *E. coli* 10B (Invitrogen), and *E. coli* S17.1λ*pir*^51^ served as the donor in conjugations. All strains were routinely grown in Luria Broth shaking at 250 r.p.m., and on Luria Agar (LA), either at 30 °C (*P. fluorescens*) or 37 °C (*E. coli*). Colonies were grown inverted at room temperature (RT, ∼22 °C) in 9 mm Petri-dishes (Nunc) containing 25 ml of Pseudomonas agar F (PAF), which is a commercial formulation of King's Medium B^52^. When necessary, antibiotics (μg ml^−1^) and agar (g l^−1^) were supplemented at the following final concentration: ampicillin (100), kanamycin (50), streptomycin (50), gentamicin (30) and agar (15). All media components were Difco-branded (BD) and chemical reagents were obtained from Sigma, or as noted otherwise.

### Evolution experiments

Overnight cultures of single isolated colonies (1.5 ml) were washed in *Pseudomonas* minimal medium (PMM)^53^ and re-suspended in 1 ml PMM by vortexing. The suspensions were either serially diluted and plated to yield 5–10 isolated colonies or spotted (10 μl) as a droplet on PAF plates, and incubated until spreading fans emerged from the edge of colonies. The spreading phenotype emerges reliably in virtually all isolated and spotted colonies over time. The specific timing of the occurrence of the spreading phenotype varies greatly among individual isolated colonies. In contrast, spotted colonies generate the spreading phenotype consistently within 10 days of inoculation. Cells from the edge of the fans were streaked out on fresh PAF plates, which always produced isolated colonies of two morphologies: mucoid like M and dry like D. Each variant was streaked out again on fresh PAF plates to confirm that the morphology is retained. All variants of the D morphotype were isolated from spreading fans of discrete isolated colonies of M, whereas the revertants were isolated from spreading fans of spotted colonies of the specified morphotype. Sampling the spreading fans emerging from D morphotype or revertant colonies again always produced isolated colonies of two morphologies: mucoid like M and dry like D.

### Preparation and numerical analyses of mixed colonies

Overnight cultures were grown and re-suspended in PMM as described above. D morphotype suspensions were repeatedly passed through a 2-ml hypodermic syringe with a 23-G needle (Terumo) to break up aggregates. Different strains were mixed in equal volumes and spotted (10 μl) on PAF plates. When necessary, the suspensions were serially diluted in PMM before mixing. To estimate the initial population size of the mixed strains, each mixture was serially diluted and plated out on PMM plates supplemented with either kanamycin or streptomycin. Upon incubating the inoculated plates for a stated period of time, colonies were harvested using a bent Pasteur pipette and re-suspended in 5 ml PMM. Aggregates were broken up as described above using a 10-ml syringe, and the suspensions were serially diluted in PMM and enumerated on PMM plates supplemented with either kanamycin or streptomycin. Mixed colonies were analysed by comparing the raw colony-forming unit (CFU) data and calculating the relative fitness (W)^54^.

### Glycosyl composition analysis

Overnight cultures of M and Pf0-1 were diluted in PBS and ∼10^5^ CFU were spread-inoculated onto PAF plates. Following 48 h of incubation at RT, the cells from each plate were washed out twice with 25 ml PBS, combined, thoroughly mixed, then pelleted by centrifugation for 30 min at 13,700*g*. Extracellular polysaccharides from the supernatant were isolated and purified^55^, and glycosyl composition analysis of the purified samples was carried out at the Complex Carbohydrate Research Center (University of Georgia, USA).

### Spatial disruption experiments

The spatial structure of colonies was disrupted by two independent treatments. In the first, colonies were incubated for 3 days and disrupted by repeated orbital mixing with a sterile plastic loop. Disrupted colonies were either left to recover for 5 additional days or disrupted daily as above over the next 4 days and imaged the following day. In the second, colonies were incubated undisturbed for 5 days and cells were physically removed with a sterile loop from the centre or between the edge and centre of colonies. This was repeated on each of the following 2 days and the colonies were imaged the following day.

### Spatial construction experiments

The spatial structure of colonies was manually constructed by two independent treatments. In the first, D colonies were incubated for 1 day and either M or M* cells were spotted (1 μl) at the edge and imaged over time. In the second, green fluorescently labelled MG or M*G cells were mixed with red fluorescently labelled DR cells and the mixed colonies were incubated for 3 days. Unlabelled M, M* or D cells were then spotted (1 μl) near the edge and imaged over time. A schematic of the second treatment is shown in Fig. 5a. The same experiment was also carried out using mixed colonies of reversely labelled MR or M*R cells with DG cells.

### Motility assay

A single isolated colony from an overnight culture was inoculated onto a motility plate using a sterile inoculation loop. Motility plates were prepared using Luria Broth or King's Medium B as the basal medium, and supplemented with 0.25% (w/v) agar. The two formulations yielded identical results. Inoculated plates were incubated at RT and imaged the following day.

### Identification of the mutation in D

Whole-genome sequencing (454 FLX) was carried out by the Washington University Genome Sequencing Center (St Louis, MO, USA). Genomic DNA from D was purified, and its sequence was analysed using established procedures^30^. A two-nucleotide (TA) deletion at the 1,269–1,270th position of the coding sequence of the *wspC* gene (Pfl01_1054) was identified. To confirm the presence of the deletion mutation, *wspC* and its flanking regions in strains M, D and Pf0-1 were PCR amplified using primers cheR1 and cheR2, and both strands of each template were sequenced using internal primers cheR1B and cheR2B. The two-nucleotide deletion was confirmed to be present in D but not in M or Pf0-1. The Phusion High-Fidelity DNA Polymerase (Finnzymes) was used in all PCR reactions described in this study or stated otherwise. All primers used in this study were obtained from Integrated DNA Technologies (Leuven, Belgium) and are listed in Supplementary Table 3. Sanger-based sequencing of both DNA strands was carried out by GENEWIZ, Inc. or Source BioScience. DNA fragments were purified using the QIAquick Kit (QIAGEN) and plasmids were extracted using the QIAprep Kit (Qiagen). All enzymes were purchased from New England Biolabs, or as noted otherwise.

### Identification of mutations in other D morphotypes

The *wsp* operon was sequenced in 20 dry variants, each isolated from a single spreading fan emerging from discrete isolated M colonies. Three overlapping sequencing templates (A, B and C) were amplified by PCR: primers wspUpstream-F and wspUpstream-R for the portion containing *wspA*, *wspB* and *wspC* genes (A), primers cheRLong1 and wsp6 for *wspD* and a part of *wspE* (B), and primers wsp3 and cheRLong2 for *wspE* and *wspF* (C). Each template was sequenced in both directions with overlapping nested primers wspUp1-wspUp8 (A), wsp1-wsp4 (B) and wsp5-wsp8 (C). Primer sequences are listed in Supplementary Table 3.

### Mutant construction

The gene splicing by overlap extension method^30^,^56^,^57^ was utilized to create mutations in M. The same two-nucleotides (TA) deleted within the *wspC* gene of D was deleted by introducing the corresponding mutations in the splicing by overlap extension primers: cheRpm5f and cheRpm5r for the 5′ fragment and cheRpm3f and cheRpm3r for the 3′ fragment. The *wspC* gene was deleted in-frame using primers cheRd5f and cheRd5r for the 5′ fragment and cheRd3f and cheRd3r for the 3′ fragment. The *wspD* gene was deleted in-frame using primers cheWd5f and cheWd5r for the 5′ fragment and cheWd3f and cheWd3r for the 3′ fragment. Both *wspC* and *wspD* genes were deleted in-frame using primers wspCDd5f and wspCDd5r for the 5′ fragment and wspCDd3f and cheWd3r for the 3′ fragment. The *Pfl01_3834* gene was deleted using primers galEd5f and galEd5r for the 5′ fragment and galEd3f and galEd3r for the 3′ fragment. Platinum Taq DNA Polymerase High Fidelity (Invitrogen) was used in the PCR reactions to facilitate the downstream T-A cloning process. The two fragments for each set were joined by PCR using the respective 5f and 3r primer pairs and cloned into the pGEM-T Easy vector system (Promega). Each mutant construct was then sub-cloned into the *Eco*RI site of the suicide plasmid pMQ30 (ref. 58; *wspC:D*, Δ*wspC*, Δ*wspD* and Δ*Pfl01_3834*) or the *Not*I site of the suicide plasmid pSR47s^57^ (Δ*wspC*Δ*wspD*). Each suicide plasmid was electroporated into *E. coli* S17.1λ*pir*, mated with M on LA plates at 30 °C for 5 h, then plated out on PMM plates supplemented with gentamicin (pMQ30) or kanamycin (pSR47s). Isolated colonies were grown overnight, serially diluted and plated out on PMM plates supplemented with 5% sucrose (w/v). Primer sets cheR-F/cheR-R, cheW1/cheW2, cheR-F/cheW2 and galE1/galE2 were used to monitor the deletion of *wspC*, *wspD*, *wspCwspD* and *Pfl01_3834*, respectively, by PCR and subsequently confirmed by sequencing both strands.

### Antibiotic resistance and fluorescent protein labelling

The mini-*Tn*7 system was used to tag the chromosomes of strains using established procedures with neutral kanamycin or streptomycin resistance cassettes^30^,^59^, and GFP or DsRedExpress proteins^30^,^60^.

### Transposon mutagenesis

Plasmid pUT-mini*Tn*5-Km*lacZ*2 (refs 61, 62) was introduced into *E. coli* S17.1λ*pir* to create the donor strain. Overnight cultures of the donor and target strains were washed in fresh PMM and mixed at a 1:1 ratio, respectively. The mixture was spotted and adsorbed on the surface of LA, incubated at 30 °C for 3 h, harvested and plated out on PMM plates supplemented with kanamycin. Approximately 6,000 transconjugants were directly screened on the selection plates for the absence of the mucoid phenotype (M served as the target strain), and ∼2,000 transconjugants were screened for the reversion to the mucoid phenotype (D served as the target strain). Transposon insertion sites were identified using established procedures by Arbitrary primed PCR, using ARB1 and ARB6 primers^63^ with the transposon-specific primer lacZext2 (ref. 61) in the first round and ARB2 (ref. 63) and lacZext1 (ref. 61) in the second round. The resulting PCR products were sequenced using lacZext1, and BLAST^64^ against the non-redundant database was used to identify the transposon insertion sites (blastn) and the homologues of the mutated loci (blastp).

### Statistical analyses

Non-parametric tests were avoided due to the small sample size (*n*=3). One-way analysis of variance (standard weighted-means) of independent samples was applied to compare multiple means, followed by pair-wise comparisons using the Tukey's honest significant difference test.

### Imaging

Epifluorescence microscopy imaging was carried out with the Axio Zoom.V16 microscope (Zeiss) under the PlanApo Z × 0.5 and × 2.3 objectives and the associated Zen Blue software. This system allows direct imaging of colonies on the agar surface without coverslips. Individual colour channels were acquired using the enhanced GFP and DsRedExpress filters in addition to a bright-field channel. Each channel image was acquired under auto-exposure to minimum and maximum thresholds. Identical linear adjustments were further applied to minimize signal bleeding between the channels across each set of images. Confocal laser scanning microscopy imaging was carried out with the LSM 700 laser scanning microscope (Zeiss) under the × 10 and × 50 objectives and the associated Zen Black software. A square piece of agar containing the entire colony was cut out and placed on slides without a coverslip for imaging. Green and red channel images were acquired separately, which prevents signal bleeding. Green channel images were acquired using 2–5% laser power and the red channel images were acquired using 5–20% laser power. Identical laser settings were used to acquire each set of images. Confocal stacks were rendered three-dimensionally using the Zen Black software. For time-lapse confocal laser scanning microscopy, colonies were directly imaged from 9 mm Petri-dishes containing 60 ml PAF. Only the green channel images were acquired at 1% laser power to minimize photo-bleaching, with the exception of the final frame, where both green and red channel images were acquired. Digital photographs of colonies were taken with the EOS 30D DSLR (Canon) or the Lumix FZ200 (Panasonic) cameras.

## Additional information

**How to cite this article:** Kim, W. *et al*. Rapid radiation in bacteria leads to a division of labour. *Nat. Commun.* 7:10508 doi: 10.1038/ncomms10508 (2016).

## Supplementary Material

Supplementary Information Supplementary Figures 1-5, Supplementary Table 1-3 and Supplementary References

Supplementary Movie 1 Time-lapse confocal imaging of the spreading edge. Movie of a 1:1 mixed colony of fluorescently labelled M (green) and D (red) captured in 10 min intervals on day 5. To limit photo-bleaching, only the green channel image was acquired with the exception of the last frame.

Supplementary Movie 2 Time-lapse confocal imaging of the area behind the spreading edge. Movie of a 1:1 mixed colony of fluorescently labelled M (green) and D (red) captured in 20 min intervals on day 4. M cells that are positioned below D cells remain stationary. To limit photobleaching, only the green channel image was acquired with the exception of the last frame.
