# Supplementary material for: Rapid radiation in bacteria leads to a division of labour
Source: Nat Commun. 2016 Feb 8;7:10508. doi: 10.1038/ncomms10508 (PMC4748119; doi:10.1038/ncomms10508)
Supplement: Supplementary Information — Supplementary Figures 1-5, Supplementary Table 1-3 and Supplementary References [file ncomms10508-s1.pdf]

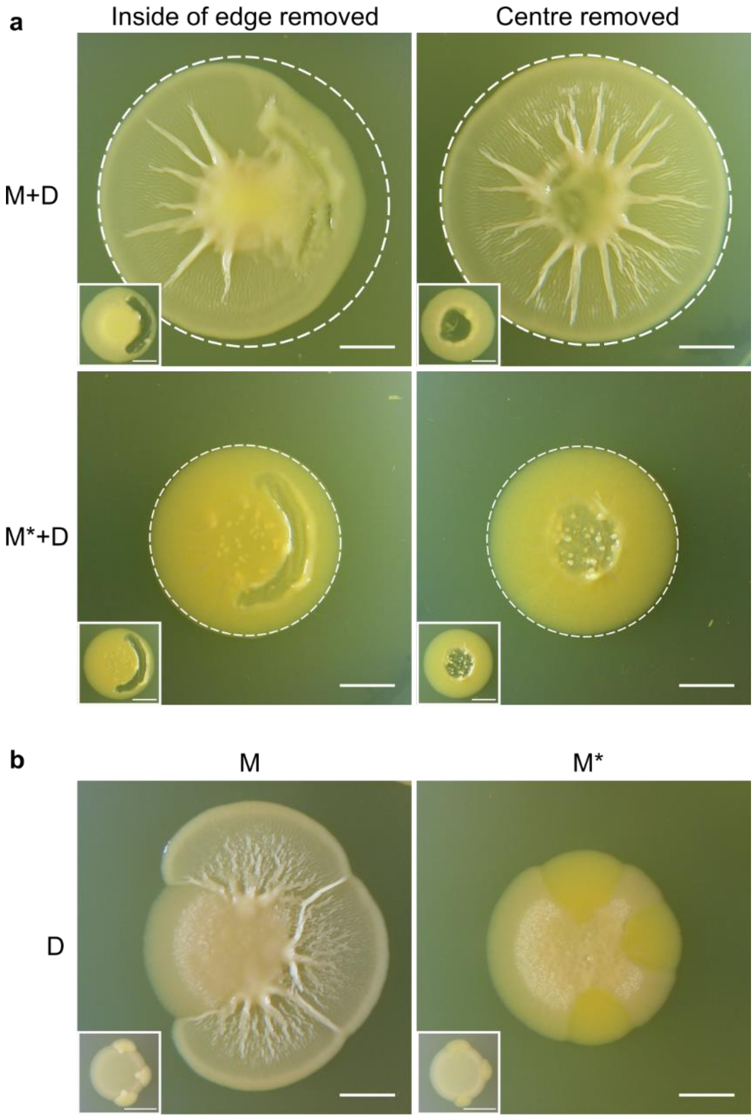

Supplementary Fig. 1. Effect of manually removing cells or constructing spatial structures on spreading. (**a**) Day 8 images of 1:1 mixed colonies wherein cells were previously removed between the edge and centre (left) or from the centre (right). The dotted circle marks the expected circumference associated with undisturbed spreading. The inset images show day 5 colonies following cell removal. (**b**) Placing M cells at the edge of a D colony produces the spreading phenotype (day 8), but M* (no mucoid polymer) cells do not. Inset images show M (left) or M* (right) cells growing at the edge of D colonies on day 2 (i.e. 24 h after spotting). Scale bars represent 5 mm.

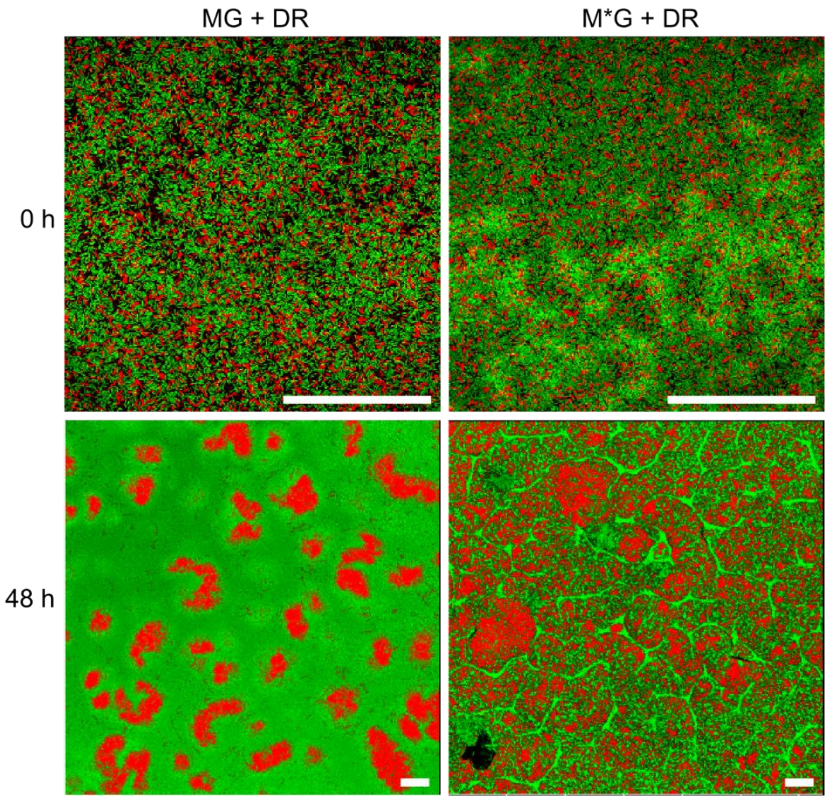

Supplementary Fig. 2. The mucoid polymer produced by M is crucial for the spatial segregation of D within mixed colonies. Confocal images of the space near the centre of 1:1 mixed colonies of fluorescently labelled D (red, DR) and M (green, MG) or M* (green, M*G). The mucoid polymer of M appears to enhance the segregation of D. Shown are 3D renderings of the confocal stacks. Scale bars represent 100 um.

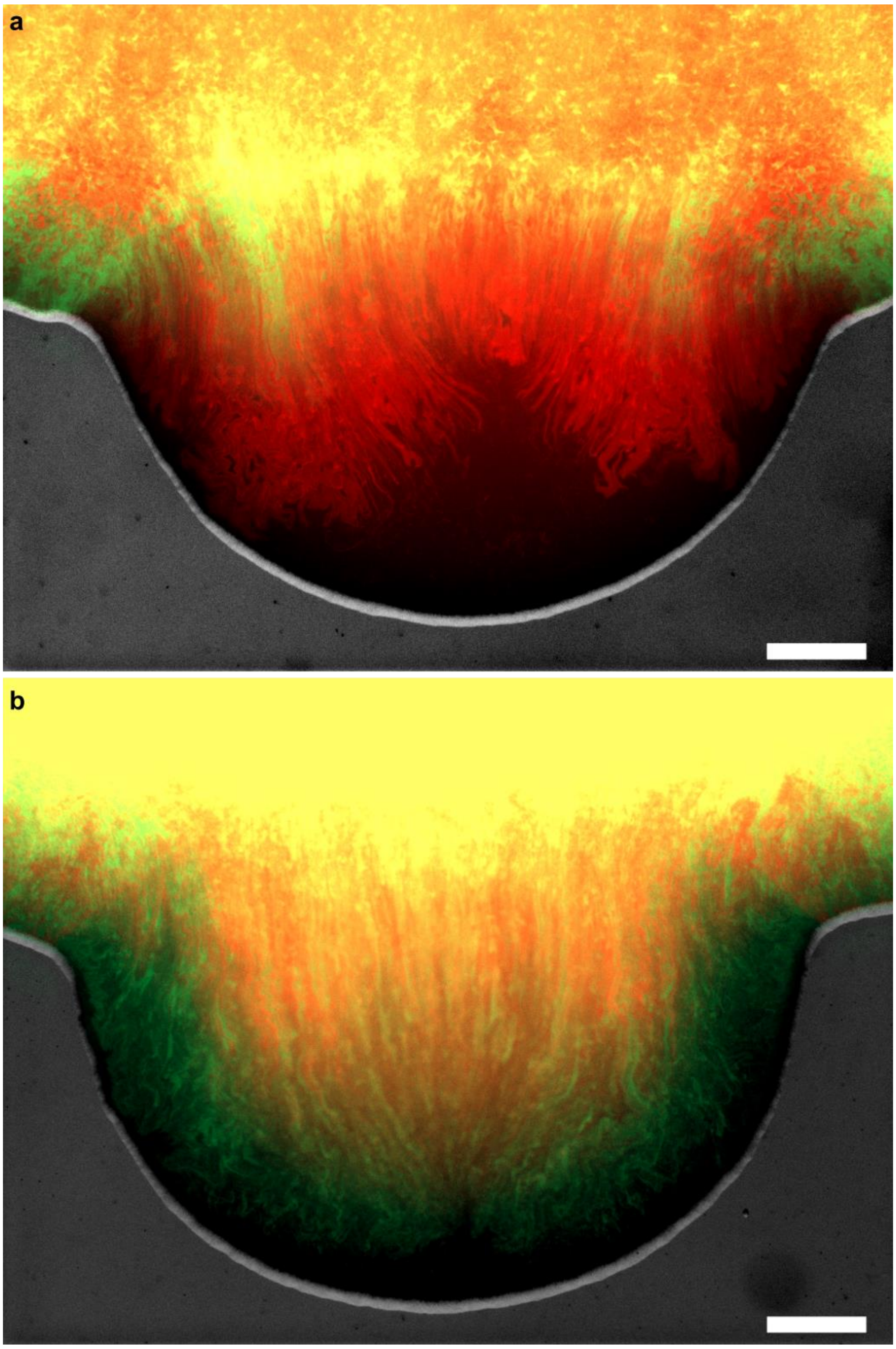

Supplementary Fig. 3. D streams into unlabelled M that is added to the spreading edge. (**a**) Epifluorescence image of the edge of a mixed colony of M (green) and D (red) on day 4, a day after placing unlabeled M cells near the edge. This is the same image as shown in Fig. 5c. (**b**) Reverse colours image of the edge of a mixed colony of M (red) and D (green) on day 4, a day after placing unlabeled M cells at the edge. In both cases, D pushes and streams into the unlabeled M before reestablishing the collective phenotype and expanding the colony edge outwards. The thresholds of the different channels in the images were linearly adjusted beyond saturation to show the spreading of D through unlabeled M. Scale bars represent 0.5 mm.

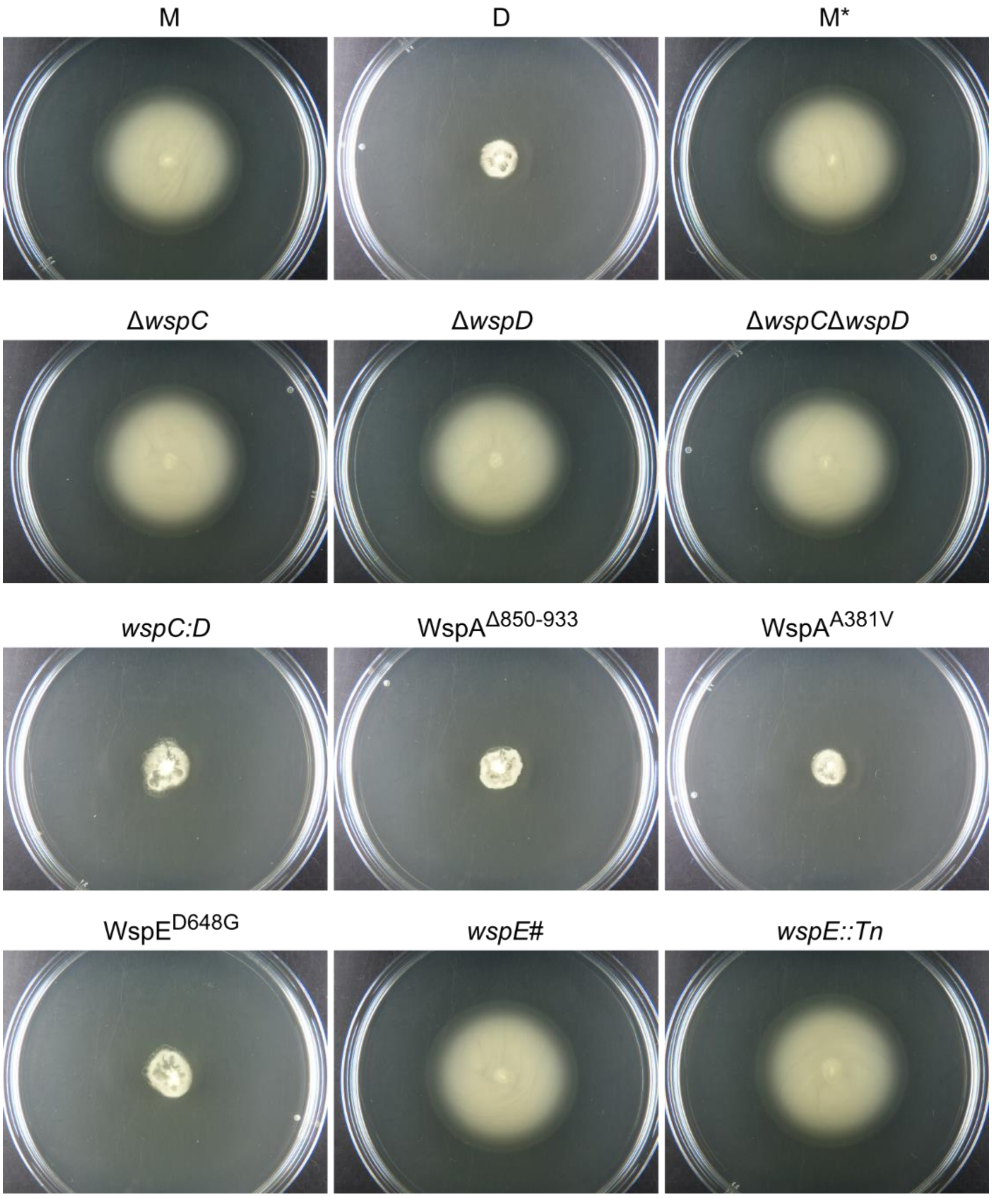

Supplementary Fig. 4. Motility phenotype of various M and D morphotypes. All strains of the M morphotype are motile while those of the D morphotype exhibit reduced motility. The diameter of each plate is 90 mm.

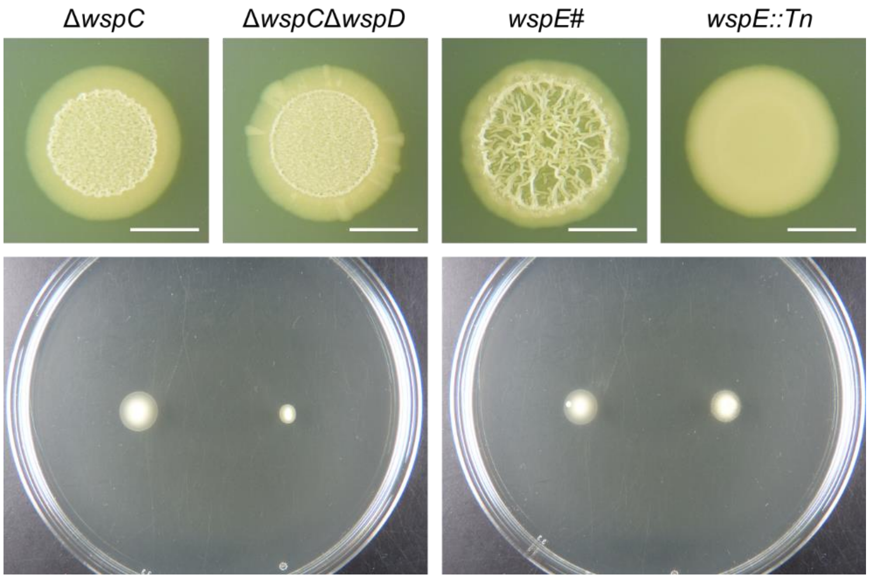

Supplementary Fig. 5. Bi-directional selection of M and D morphotypes. Day 3 images of D morphotype strains isolated from various M morphotype parent strains (top). Scale bars represent 5 mm. Shown below is the motility phenotype of the corresponding strains from the top panels. The diameter of each plate is 90 mm.

Supplementary Table 1. Glycosyl composition analysis of extracellular polysaccharides produced by M and Pf0-1

Sample	Glycosyl residue	Mass (ug)[a]	Mole %[b]
M	Rhamnose	n.d.	n.d.
	Xylose	n.d.	n.d.
	Glucuronic acid	n.d.	n.d.
	Galacturonic acid	n.d.	n.d.
	Mannose	n.d.	n.d.
	Galactose	5.6	3.6
	Glucose	150.6	96.4
	N-acetyl galactosamine	n.d.	n.d.
	N-acetyl glucosamine	n.d.	n.d.
	Heptose	n.d.	n.d.
	3-deoxy-2-manno-2-octulsonic acid	n.d.	n.d.
	Sum	156.2	100
Pf0-1	Rhamnose	0.4	1.7
	Xylose	1.0	5.0
	Glucuronic acid	1.3	4.8
	Galacturonic acid	n.d.	n.d.
	Mannose	0.6	2.3
	Galactose	8.7	35.6
	Glucose	12.4	50.5
	N-acetyl galactosamine	n.d.	n.d.
	N-acetyl glucosamine	n.d.	n.d.
	Heptose	n.d.	n.d.
	3-deoxy-2-manno-2-octulsonic acid	n.d.	n.d.
	Sum	24.4	100

[a] n.d. denotes none detected.

[b] Values are expressed as mole percent of total carbohydrate.

Supplementary Table 2. Description of *Pseudomonas fluorescens* strains used in this study

Strain	Genotype[a]	Phenotype	Reference
Pf0-1	Wildtype	Wildtype	[1]
Pf0-1MV (M)	Pf0-1 (*rsmE*Δ126)	Mucoid	[2]
MK	Pf0-1MV (*Tn7*-Km)	Mucoid	Constructed isolate
MS	Pf0-1MV (*Tn7*-Sm)	Mucoid	Constructed isolate
MG	Pf0-1MV (*Tn7*-Gm(*gfp2*))	Mucoid	Constructed isolate
MR	Pf0-1MV (*Tn7*-Gm(DsRedExpress-b))	Mucoid	Constructed isolate
Pf0-1DV (D)	Pf0-1MV (*wspC*Δ1269-70)	Dry	Evolved isolate
DK	Pf0-1DV (*Tn7*-Km)	Dry	Constructed isolate
DS	Pf0-1DV (*Tn7*-Sm)	Dry	Constructed isolate
DG	Pf0-1DV (*Tn7*-Gm(*gfp2*))	Dry	Constructed isolate
DR	Pf0-1DV (*Tn7*-Gm(DsRedExpress-b))	Dry	Constructed isolate
M*	Pf0-1MV (Δ*Pfl01_3834*)	Non-mucoid	Constructed isolate
M*K	M* (*Tn7*-Km)	Non-mucoid	Constructed isolate
M*G	M* (*Tn7*-Gm(*gfp2*))	Non-mucoid	Constructed isolate
M*::Tn1	Pf0-1MV (*Pfl01_3834*^{44}*::Tn5*)	Non-mucoid	Constructed isolate
M*::Tn2	Pf0-1MV (*Pfl01_3834*^{257}*::Tn5*)	Non-mucoid	Constructed isolate
M*::Tn3	Pf0-1MV (*Pfl01_3839*^{502}*::Tn5*)	Non-mucoid	Constructed isolate
WspA$^{\Delta 850\text{-}933}$	Pf0-1MV (*wspA*Δ850-933)	Dry	Evolved isolate
WspA^{A381V}	Pf0-1MV (*wspA*^{C}1142^{T})	Dry	Evolved isolate
wspC:D	Pf0-1MV (*wspC*Δ1269-70)	Dry	Constructed isolate
Δ*wspC*	Pf0-1MV (Δ*wspC*)	Mucoid	Constructed isolate
Δ*wspD*	Pf0-1MV (Δ*wspD*)	Mucoid	Constructed isolate
Δ*wspC*Δ*wspD*	Pf0-1MV (Δ*wspC* Δ*wspD*)	Mucoid	Constructed isolate
WspE^{D648G}	Pf0-1MV (*wspE*^{A}1943^{G})	Dry	Evolved isolate
wspE#	WspA$^{\Delta 850\text{-}933}$ (*wspE*^{C}134^{A})	Mucoid	Evolved isolate
wspE::Tn	Pf0-1DV (*wspE*^{1360}*::Tn5*)	Mucoid	Constructed isolate

[a] Δ denotes the relative position(s) of nucleotide deletion within the coding sequence (CDS); substitution mutation is indicated at the specific position of the CDS (^{original}position^{substitution}); *Tn5* denotes the mini transposon *Tn5-KmlacZ2*, and the insertion site is indicated as the nucleotide position within the CDS (gene^{position}::Tn5); *Tn7* denotes the mini transposon *Tn7* harboring different antibiotic resistance cassettes and constitutively expressed fluorescent proteins as indicated.

Supplementary Table 3. Description of PCR primers used in this study

Primers	Sequence
cheR1	CAACCGTCCGTTTGCTGGCG
cheR1B	AAAAGCACAAATCCCGACGC
cheR2	GCAACGCATAGCGGTCGAGC
cheR2B	AATTGCGGCAATGGATGTGC
cheR-F	AGTCTGATGAGCGAAATCACCG
cheR-R	CTCGTCGATGGCGTGAATCC
cheRpm5f	CGTCACAGCGAACCGCAGC
cheRpm5r	GTGTCGGACGGGATCATCGTTCGCTCACTGTCG
cheRpm3f	GCACCGCCGACAGTGAGCGAACGATGATCCCGT
cheRpm3r	GCCGCGATGATCAGCATGC
cheRd5f	CGAAGCAGGCGTTGTTTCTG
cheRd5r	CGTTCAAGGTGTCGGACGGGATCATGGCTGCTC
cheRd3f	GGCTTGAGGAGCAGCCATGATCCCGTCCGACAC
cheRd3r	GGCCGCGATGATCAGCATGC
cheRLong1	GCCAGACGATTGCAGGAGCG
cheRLong2	TCATCGCCTGATCGTCCACC
cheW1	AAGCGCCTGACCCATGTCGATG
cheW2	TTCAGGCGTTCGGCGGTGAC
cheWd5f	CAGTCGTTCGCCTTCAGTCG
cheWd5r	CGCATTTGCTCGGGGGTCATCGTTTACGCTCACT
cheWd3f	GCCGACAGTGAGCGTAAACGATGACCCCCGAGC
cheWd3r	GTGGTGCGTTTGCTCTTGCG
galE1	TGACGATGGCCATGTGTTGCAGG
galE2	GCCGGGGGAGAACATGCAAC
galEd5f	CGTCCCGTGCTTCTTCAACG
galEd5r	CTAACATTGCACTAAAACGCCATCAAGTGGGAA
galEd3f	CGAGTTGGCACTTTTCCCACTTGATGGCGTTTTA
galEd3r	TCCAAAAACAAAGTCACCCG
wspCD5f	GGTGGCGAAGCAGGCGTTGT
wspCD5r	CGCATTTGCTCGGGGGTCATGGCTGCTCCTCAAG
wspCD3f	CGCGGCTTGAGGAGCAGCCATGACCCCCGAGCA
wsp1	GCCGACAGTGAGCGTAAACG
wsp2	ATCAGCAAATCGGTGCCCTG
wsp3	GGTTGTTCCTGCGTCCGGAG
wsp4	CTCTTCACTCAACTGGGCGGC
wsp5	CGGCAATGGTGTGGATCTGG
wsp6	CATGAAAACTGGCCTTGGCC
wsp7	AAGATCGCCGCCGTGGACTG
wsp8	AACACCATGGCGGCGTTCTC
wspUpstream-F	CTGTTGTCAGCGATATCAGAACCCG
wspUpstream-R	CCGATGCGGTTCCAGCAATC
wspUp1	CCGATTGCCGGAAAGTCCCAG
wspUp2	TACGAGATCGCGGGAGGTCG
wspUp3	CGACCTCCAAGCAACAGCAAG
wspUp4	GGCACTGAGGTCGATCACCG
wspUp5	CCTGCCGTTGAAGCCGATTG
wspUp6	CGCGGACGCTTTCACTCACC
wspUp7	TCGATGGCATGGACGTCAGC
wspUp8	TTCGTGGGTGACGTTCAAGG

Supplementary References

1. Compeau, G., Al-Achi, B. J., Platsouka, E. & Levy, S. B. Survival of rifampin-resistant mutants of Pseudomonas fluorescens and Pseudomonas putida in soil systems. *Appl. Environ. Microbiol.* **54,** 2432–8 (1988).

2. Kim, W., Racimo, F., Schluter, J., Levy, S. B. & Foster, K. R. Importance of positioning for microbial evolution. *Proc. Natl. Acad. Sci. U. S. A.* **111,** E1639–47 (2014).
